# Complete Genome Sequencing of *Elizabethkingia* sp. Strain 2-6

**DOI:** 10.1128/MRA.00805-19

**Published:** 2019-10-31

**Authors:** Kuan-Ming Liu, Hui-Lan Chang, Ming-Hong Hsu, Yi-Zhen Lin, Yu-Lin Lee, Ying-Tsong Chen

**Affiliations:** a Institute of Genomics and Bioinformatics, National Chung Hsing University, Taichung, Taiwan; b Department of Infection, Changhua Christian Hospital, Changhua, Taiwan; c Institute of Molecular and Genomic Medicine, National Health Research Institutes, Zhunan, Taiwan; Indiana University, Bloomington

## Abstract

Elizabethkingia sp. strain 2-6 was collected from a water faucet in the intensive care unit of a medical center in Taiwan. The complete genome sequence and annotation are reported. Analysis of the genetic relatedness to the known *Elizabethkingia* genomes indicated that strain 2-6 may be a new genomospecies of *Elizabethkingia*.

## ANNOUNCEMENT

Elizabethkingia is a genus of highly resistant, Gram-negative bacilli that is ubiquitous in the environment. The genus contains six species, namely, E. meningoseptica, E. miricola, E. anophelis, E. bruuniana, E. ursingii, and E. occulta (1–3). They are phenotypically very similar, sometimes leading to misidentifications in clinical laboratories (4). Nosocomial infections caused by *Elizabethkingia* spp. are an emerging problem in Taiwan. The majority of the infections in Taiwan have been meningitis outbreaks in premature newborns or infants. Nosocomial infections caused by *Elizabethkingia* spp. in adult patients are rare, and most cases have been reported in Taiwan (5). The cases of infections caused by *Elizabethkingia* spp. in Changhua Christian Hospital (CCH), a medical center in central Taiwan, began to soar since 2015, from 1 to 3 cases a month in 2000 to 12 cases a month in 2017. Almost all of the cases were from the respiratory care unit (RCU) and intensive care unit (ICU) of the hospital. In a surveillance project carried out in CCH from 2016 to 2018, *Elizabethkingia* isolates from clinical samples and the environment of the RCU/ICU were collected for analysis.

*Elizabethkingia* sp. strain 2-6 was isolated from a swab sample from one of the water faucets in the ICU. Bacteria from the swab were first cultured in thioglycolate broth for 48 h at 35°C and then transferred to a blood agar plate/eosin methylene blue (BAP/EMB) agar plate for another 24 h at 35°C. The strain was identified either as *Elizabethkingia* species or *E. meningoseptica* using the API20NE system (bioMérieux), Vitek mass spectrometer (MS) (bioMérieux), and Bruker MALDI Biotyper (BD). In an attempt to determine the genetic relatedness of the *Elizabethkingia* isolates from the ICU, pulsed-field gel electrophoresis (PFGE) analysis was performed on the bacterial genomic DNA samples digested by the restriction endonuclease XhoI. Interestingly, strain 2-6 was found to have a PFGE pattern different from the rest of those of the *Elizabethkingia* isolates we collected in the hospital.

Sequencing of the genome was carried out using the Illumina iSeq 100 and Nanopore MinION platforms. For short-read sequencing, the genomic DNA of *Elizabethkingia* sp. strain 2-6 was prepared using the DNeasy UltraClean microbial kit (Qiagen). Sequencing was carried out using an iSeq 100 sequencer. The Nextera DNA Flex kit (Illumina) and barcode kit were used for shotgun library generation, following standard protocols. Sequencing of the library generated 150-bp paired-end reads, followed by adapter trimming using the standard pipeline provided by the vendor. This generated a total of 1,343,458 reads with a total of 198.5 Mbp (∼45× coverage). For Nanopore sequencing, the genomic DNA was purified from the cultured bacteria using a DNeasy blood and tissue kit (Qiagen). The sequencing library was prepared using a rapid 1D sequencing kit with barcoding, following standard protocols. Sequencing was performed using a Nanopore MinION device. Reads were called using Guppy version 2.3.7 (Nanopore). Only the reads that passed the initial quality check (QC) were used. This generated 31,175 reads for a total of 320 Mbp (∼72.8× coverage). The maximum, *N*_50_, and average read lengths are 115 kb, 17.4 kb, and 11 kb, respectively.

Hybrid assembly of the Illumina short paired-end reads and Nanopore long reads was performed using Unicycler version 0.4.8 (6), which resulted in a single circular chromosome of 4,394,713 bp, with an average G+C content of 35.86%. The correctness of the assembly result was rechecked by using another assembler, Canu 1.8 (7), with the aid of CLC Genomics Workbench 11 (Qiagen). The genome sequence was annotated using the NCBI Prokaryotic Genome Annotation Pipeline (PGAP) (8). The genome of *Elizabethkingia* sp. 2-6 is predicted to contain 4,038 genes, of which 3,910 are protein-coding genes. There are 73 RNA genes, including 15 rRNA (5 each of 5S, 16S, and 23S rRNA), 55 tRNA, and 3 noncoding RNA genes.

Interestingly, average nucleotide identity (ANI) analysis (9) resulted in ANI scores of <95% between strain 2-6 and the 6 known species of the *Elizabethkingia* genus (ANI score, 79.86% for *E. meningoseptica* G4120 [GenBank accession number NZ_CP016378], 92.78% for *E. miricola* BM10 [GenBank accession number NZ_CP011059], 91.67% for *E. anopheles* R26 [GenBank accession number NZ_CP023401], 92.63% for *E. bruuniana* G0146 [GenBank accession number NZ_CP014337], 90.96% for *E. ursingii* G4123 [GenBank accession number NZ_CP016377], and 90.81% for *E. occulta* G4070 [GenBank assembly number GCA_002023715]). The results suggest that strain 2-6 may be another new addition to the *Elizabethkingia* genus.

### Data availability.

This complete genome sequence of *Elizabethkingia* sp. 2-6 has been deposited at GenBank under the accession number NZ_CP039929 and BioProject number PRJNA540378. The sequencing reads have been deposited in the NCBI Sequence Read Archive (SRA) under the accession numbers SRR9643623 and SRR9643624.
